# Treatment Non-adherence Patterns Among Patients With Mental Illness: A Study From the District Mental Health Care Center in India

**DOI:** 10.7759/cureus.54495

**Published:** 2024-02-19

**Authors:** Jahangir Khan, Jwaad A Khan, Subhra Kumari, Deepak Charan

**Affiliations:** 1 Department of Healthcare and Pharmaceutical Management, School of Management and Business Studies, Jamia Hamdard, New Delhi, IND; 2 Department of Pathology, Shri Ram Murti Smarak Institute of Medical Sciences, Bareilly, IND; 3 Department of Psychiatry, Shri Ram Murti Smarak Institute of Medical Sciences, Bareilly, IND

**Keywords:** treatment non-compliance, district mental health care center, non-adherence pattern, treatment discontinuation, mental illness

## Abstract

Introduction: The success of any medical intervention, including mental health treatment, depends largely on patient adherence to the prescribed regimen. In psychiatric illnesses, one of the biggest problems is getting people to adhere to their treatment schedule, representing a treatment gap that increases the burdens of patients, families, communities, and countries. Globally, it has become necessary for community health organizations to actively work towards reducing this gap and treatment non-adherence. Therefore, in this study, we aimed to examine treatment non-adherence patterns among patients with mental illness.

Materials and methods: This work used a retrospective study design and consecutive sampling. The data source was secondary data obtained from the healthcare records of patients registered in the outpatient department of the District Mental Health Care Center, India, from January 2022 to December 2022.

Results: Out of a total of 883 patients recruited for the study, 35.7% (n=315) were on regular follow-up over a duration of more than one year. Among patients with severe mental illness, 46% (n=46) had regular follow-ups and were compliant with therapy. About 49% of patients (n=433) discontinued their treatment after the initial contact with the therapist, with the highest rate among those with substance use disorders (77.0%; n=57). The remaining 15.3% (n=135) of recruited patients discontinued their follow-up appointments over a duration of 1 week to 12 months. Overall, 64.3% (n=568) of the recruited patients discontinued their treatment within one year.

Conclusion: There was considerable early treatment dropout among patients with mental illness. However, this treatment discontinuation can be avoided because the individual identities of these patients are well-known to the therapist or facility, as they have had at least one interaction with the therapist. In order to improve treatment adherence, patients with mental illnesses must receive consistent support through community outreach programs, home visits, and new strategies to promote treatment compliance.

## Introduction

Non-adherence to treatment among patients with mental illness is a common problem globally. The relationship between mental illnesses and dropout from treatment is still a subject of considerable debate. Although mental illness accounts for 11.8% of the overall disease burden in India [1], only 10% of those currently suffering from mental illness are thought to be receiving evidence-based treatments [2]. According to the National Mental Health Survey of India (2016), the lifetime prevalence of mental illness is 13.67%, with a treatment gap of 84.5% for any mental illness [3].

Various studies show that 20%-50% of patients are at least partially not in compliance with a treatment plan, and these percentages increase to 70%-80% for patients with mental illnesses [4]. Mental illnesses require long-term follow-up care services to avoid relapse and enhance the individual’s overall well-being [5]. However, the rate of early treatment dropout among patients with mental illnesses who seek treatment is high, particularly among the adult patient population. Various research findings suggest that 30%-50% of patients who attend their first therapy session never return for their next follow-up [6].

Some have defined non-adherence as “ending therapy without the provider’s consent, regardless of the number of sessions” or “failure to adhere to follow-up after an introductory interview” [7,8].

Patients with mental illness and without caregivers are frequently left without treatment, while patients who have families or caregivers and are seeking assistance commonly switch to non-psychiatric practitioners, including practitioners of Indian conventional medicine, spiritual healers, astrologers, and faith-based healers [9]. Mental illnesses that go untreated or are treated inconsistently may result in poor quality of life, physical or mental impairment, low income, marriage separation, homelessness, low income, absenteeism from work, domestic abuse, substance abuse, or increased caregiver burden [10]. Additionally, it has been demonstrated that non-compliance is linked to higher rates of unintentional admissions, prolonged hospitalizations, increased mortality, poor prognosis, and poor quality of life [11-13].

Previous studies from India have found varying percentages of treatment non-compliance among different mental health conditions. In one study, out of a total of 528 patients, 29.7% were receiving regular follow-up with behavioral health services for a duration of 12 consecutive months or more. A large percentage of the patients (36.2%) dropped out of therapy after their first visit and 34.1% terminated subsequent visits between the 1st week and the 12th month of follow-up [5]. Previous studies showed that approximately 30%-60% of patients with depressive disorders discontinued their medical treatment without the consent of their doctor [14,15]. In a study conducted by Banerjee and Varma, out of a total of 239 patients with unipolar depressive disorder, 66.9% (n=160) were non-adherent and 33.1% (n=79) were adherent to therapy [16].

According to research by Australia’s Headspace model of care, it was estimated that 30%-75% of young people who are able to access psychological services will stop therapy early [17]. Demyttenaere et al., estimated that among those living in low- and middle-income countries, 75%-85% of persons with serious mental health issues are not able to access the appropriate medical treatment that they need, in contrast to 35%-50% of those in high-income countries [18]. In another study, 38.2% of patients stopped receiving therapy following their first appointment, and, of the remaining individuals, 61.8% stopped receiving therapy within a period of six months [19]. Another study found that approximately a quarter of patients discontinued treatment shortly after their initial appointment [20]. Studies have shown that >50% of individuals diagnosed with schizophrenia fail to adhere to their treatment plan [21-23].

Previous studies have found various sociodemographic and clinical factors that contribute to non-adherence, including male gender, younger age, joblessness, poor socioeconomic status, previous history of non-compliance with therapy, history of using various substances, severity of illness, impaired insight, and cognitive impairment. Treatment-related factors that contribute to non-adherence include treatment ineffectiveness, adverse effects, and a complex treatment schedule [23-27]. Other factors have been shown to influence treatment adherence, such as stigma associated with mental illnesses, differing beliefs among patients and caregivers regarding the etiology of illness (e.g., supernatural activity or stressful life circumstances), familial support, and ease of access to healthcare facilities [28,29].

Adherence to treatment is an important challenge for mental health service providers, as non-adherence leads to overall poor prognosis, increased health care burden, and poor quality of life. Despite this challenge, very few studies have been carried out on Indian populations suffering from various mental illnesses and treatment non-adherence. Therefore, this study was conducted to explore the general pattern of treatment non-adherence among individuals diagnosed with mental illness at the District Mental Health Care Center, India.

## Materials and methods

Study settings, design, and sampling

This study was conducted at the District Mental Health Care Center (DMHC), situated in the Nuh district in India. It is a central government-funded center that provides mental health care services to approximately 100 villages located within the catchment geographical area, covering a radius of approximately 40-45 km (i.e., approximately every village in the district) covering a population of approximately 5-6 lakhs. The psychiatry outpatient department at the facility is generally open six days per week. The center is supported by a multi-disciplinary clinical staff, including a psychiatrist, a psychologist, a social worker, a psychiatric nurse, and other staff members responsible for administrative responsibilities. Both assessments and interventions are provided free of charge with a minimal registration fee of Rs. 5 (approximately 6 cents USD). The hospital dispensary provides medications to patients free of charge.

In this retrospective observational study, consecutive sampling was adopted, and secondary data were obtained from the healthcare records of patients registered in the outpatient department of the DMHC from January 2022 to December 2022.

Data collection

A retrospective file review was performed on 1190 patient health records, among which 307 files were rejected due to the absence of an International Classification of Diseases-10 (ICD-10) diagnosis, primary diagnosis of headache or mental retardation, or visiting the center only to obtain a mental disability certificate. In this study, mental illnesses were classified based on the system provided by ICD-10 of the World Health Organization, the categories included severe mental disorders (SMDs), common mental disorders (CMDs), substance use disorders (SUDs), and intellectual developmental disorders (IDDs), as presented in Table 1 [5].

**Table 1:** Categorization of mental illnesses ICD-10: International Classification of Diseases-10

S. No.	ICD-10 code	Classification of ICD	Categorization of disorders
1	F10-F19	Mental and behavioral disorders due to psychoactive substance use disorders	Substance use disorders (SUDs)
2	F20-F29 F30-F31	Schizophrenia, schizotypal and delusional disorders, mood (affective) disorders	Severe mental disorders (SMDs)
3	F32-F39 F40-F48	Depressive disorders, neurotic, stress-related and somatoform disorders	Common mental disorders (CMDs)
4	F70-F79 F80-F89	Mental retardation disorder of psychological development	Intellectual developmental disorders (IDDs) + others

Treatment adherence and non-adherence

In this study, treatment adherence is considered consistent attendance of patients at scheduled appointments with the DMHC, with no more than two consecutive appointments missed within a 12-month period. In contrast, treatment non-adherence is considered a treatment dropout resulting in patients failing to attend any subsequent follow-up appointments at the DMHC within a 12-month period following their initial visit. Therapeutic non-adherence, alternatively referred to as dropout, cessation, discontinuation, termination, disengagement, or inconsistent follow-up, encompasses many terms denoting the lack of adherence to a prescribed treatment regimen.

Ethical clearance

The Jamia Hamdard Institutional Ethics Committee, New Delhi, India, granted ethical clearance for the study, and permission was obtained from the chief medical officer of the concerned DMHC. Data collection began after obtaining the ethical clearance. Informed consent was not necessary from the participants as this was a retrospective study, and patient anonymity has been maintained.

Statistical analysis

The descriptive statistics percentages and measures of central tendency were calculated and data were assessed for normal distribution. The sociodemographic characteristics of the study participants were analyzed using descriptive statistics. Continuous variables were described as mean and standard deviation, while categorical variables were described as percentages and proportions. The IBM SPSS Statistics for Windows, Version 29 (Released 2021; IBM Corp., Armonk, New York, United States) was used for data analysis.

## Results

Out of the total recruited population (n=883), 93.10% (n=822) were Muslim. The majority of the patients (60.6%; n=535) were male, and the minority were female (39.4%; n=348). In accordance with diagnosis-specific classification, SUDs (94.6%; n=70), SMDs (76.0%; n=76), and CMDs (56.4%; n=308) were more prevalent among male patients, whereas the rate of IDDs was almost equal among both genders (i.e., 50.3%; n=82 female and 49.7%; n=81 male). Among all diagnoses, CMDs (61.8%; n=546) were predominant, followed by IDDs (18.5%; n=163) and SMDs (11.3%; n=100). About 8.4% (n=74) of patients had a primary diagnosis of SUDs (Table 2).

**Table 2:** Number (n) and percentage (%) distribution of mental illnesses and gender CMDs: Common mental disorders; IDDs: Intellectual developmental disorders; SMDs: Severe mental disorders; SUDs: Substance use disorders

Variable	CMDs	IDDs	SMDs	SUDs	Total
Gender	n (%)	n (%)	n (%)	n (%)	n (%)
Female	238 (43.6%)	82 (50.3%)	24 (24.0%)	4 (5.4%)	348 (39.4%)
Male	308 (56.4%)	81 (49.7%)	76 (76.0%)	70 (94.6%)	535 (60.6%)
Total	546 (61.8%)	163 (18.5%)	100 (11.3%)	74 (8.4%)	883 (100.0%)

The frequency of regular follow-up in males was 36.1% (n=193) while in females it was 35.1% (n=122), and, after the initial visit, the male dropout rate was 47.9% (n=256) while in females it was 50.9% (n=177) (Table 3).

**Table 3:** Gender and frequency of dropout

	Drop out after							
Variable	1st visit	1st follow-up	2nd follow-up	3rd follow-up	4th follow-up	6-12 months	Regular	Total
Gender	n (%)	n (%)	n (%)	n (%)	n (%)	n (%)	n (%)	n (%)
Female	177 (50.9%)	1 (0.3%)	20 (5.7%)	4 (1.1%)	9 (2.6%)	15 (4.3%)	122 (35.1%)	348 (39.4%)
Male	256 (47.9%)	5 (0.9%)	30 (5.6%)	14 (2.6%)	12(2.2%)	25 (4.7%)	193 (36.1%)	535 (60.6%)
Total	433 (49.0%)	6 (0.7%)	50 (5.7%)	18 (2.0%)	21 (2.4%)	40 (4.5%)	315 (35.7%)	883 (100.0%)

In this study, the mean age of the patients was 32.8 years (standard deviation ± 15.4). Among, the SUDs category, the highest prevalence of SUDs was found in those aged 31-40 years (15.2%; n=28), whereas among, the SMDs category, the highest prevalence of SMDs was found in those in those aged ≥61years (21.8%; n=12). As per the analysis, those aged 1-10 years had the highest prevalence of IDDs (94.3%; n=33), followed by those aged 11-20 years (49.3%; n=75); and those aged 41-50 years had the highest prevalence of CMDs (85.3%; n=87), followed by those aged 51-60 years (73.0%; n=54). Psychiatric disorders were most prevalent in those aged 21-30 years (31.8%; n=281) and 31-40 years (20.8%; n=184) (Table 4).

**Table 4:** Age-wise prevalence of mental illnesses CMDs: Common mental disorders; IDDs: Intellectual developmental disorders; SMDs: Severe mental disorders; SUDs: Substance use disorders

	CMDs	IDDs	SMDs	SUDs	Total
Age group (years)	n (%)	n (%)	n (%)	n (%)	n (%)
1-10	0 (0.0%)	33 (94.3%)	2 (5.7%)	0 (0.0%)	35 (4.0%)
11-20	54 (35.5%)	75 (49.3%)	12 (7.9%)	11 (7.2%)	152 (17.2%)
21-30	192 (68.3%)	35 (12.5%)	34 (12.1%)	20 (7.1%)	281 (31.8%)
31-40	121 (65.8%)	9 (4.9%)	26 (14.1%)	28 (15.2%)	184 (20.8%)
41-50	87 (85.3%)	3 (2.9%)	5 (4.9%)	7 (6.9%)	102 (11.6%)
51-60	54 (73.0%)	7 (9.5%)	9 (12.2%)	4 (5.4%)	74 (8.4%)
61 and above	38 (69.1%)	1 (1.8%)	12 (21.8%)	4 (7.3%)	55 (6.2%)
Total	546 (61.8%)	163 (18.5%)	100 (11.3%)	74 (8.4%)	883 (100.0%)

Out of a total of 883 patients, only 35.7% (n=315) were consistent with treatment over a duration of more than one year. The regular treatment adherence rates in patients with CMDs, IDDs, SMDs, and SUDs were 34.6% (n=189), 41.7% (n=68), 46.0% (n=46), and 16.2% (n=12), respectively. A large percentage of patients (49.0%; n=433) discontinued their treatment after the initial visit, and that percentage was highest among those with SUDs (77.0%; n=57), followed by those with CMDs (50.0%; n=273), and those with IDDs (41.1%; n=67). Among the remaining patients, 15.3% (n=135) discontinued their follow-up appointments between 1 week and 12 months of treatment. Overall, the data indicate that the dropout rate was very high after the initial visit, as 64.3% (n=568) of patients discontinued their treatment within one year (Table 5).

**Table 5:** Categories of mental illnesses and frequency of dropout CMDs: Common mental disorders; IDDs: Intellectual developmental disorders; SMDs: Severe mental disorders; SUDs: Substance use disorders

	Drop out after							
	1st visit	1st follow-up	2nd follow-up	3rd follow-up	4th follow-up	6-12 months	Regular	Total
Mental illness	n (%)	n (%)	n (%)	n (%)	n (%)	n (%)	n (%)	n (%)
CMDs	273 (50.0%)	4 (0.7%)	35 (6.4%)	13 (2.4%)	10 (1.8%)	22 (4.0%)	189 (34.6%)	546 (61.8%)
IDDs	67 (41.1%)	0 (0.0%)	9 (5.5%)	2 (1.2%)	6 (3.7%)	11 (6.7%)	68 (41.7%)	163 (18.5%)
SMDs	36 (36.0%)	1 (1.0%)	5 (5.0%)	2 (2.0%)	4 (4.0%)	6 (6.0%)	46 (46.0%)	100 (11.3%)
SUDs	57 (77.0%)	1 (1.4%)	1 (1.4%)	1 (1.4%)	1 (1.4%)	1 (1.4%)	12 (16.2%)	74 (8.4%)
Total	433 (49.0%)	6 (0.7%)	50 (5.7%)	18 (2.0%)	21 (2.4%)	40 (4.5%)	315 (35.7%)	883 (100.0%)

The frequency of regular follow-ups in the age categories of 1-10 years, 11-20 years, 21-30 years, 31-40 years, 41-50 years, 51-60 years, 61 years and above were 31.4% (n=11), 34.2% (n=52), 38.8% (n=109), 38.0% (n=70), 31.4% (n=32), 29.7% (n=22), and 34.5% (n=19), respectively. The rate of discontinuation after the first encounter was highest among the age groups of 41-50 years (52.9%; n=54) and 51-60 years (52.7%; n=39) (Table 6).

**Table 6:** Age categories and frequency of dropout

	Drop out after							
	1st visit	1st follow-up	2nd follow-up	3rd follow-up	4th follow-up	6-12 months	Regular	Total
Age group (years)	n (%)	n (%)	n (%)	n (%)	n (%)	n (%)	n (%)	n (%)
1-10	18 (51.4%)	0 (0.0%)	3 (8.6%)	0 (0.0%)	2 (5.7%)	1 (2.9%)	11 (31.4%)	35 (4.0%)
11-20	79 (52.0%)	0 (0.0%)	8 (5.3%)	1 (0.7%)	4 (2.6%)	8 (5.3%)	52 (34.2%)	152 (17.2%)
21-30	130 (46.3%)	2 (0.7%)	11 (3.9%)	5 (1.8%)	7 (2.5%)	17 (6.0%)	109 (38.8%)	281 (31.8%)
31-40	86 (46.7%)	1 (0.5%)	9 (4.9%)	7 (3.8%)	4 (2.2%)	7 (3.8%)	70 (38.0%)	184 (20.8%)
41-50	54 (52.9%)	1 (1.0%)	8 (7.8%)	3 (2.9%)	1 (1.0%)	3 (2.9%)	32 (31.4%)	102 (11.6%)
51-60	39 (52.7%)	0 (0.0%)	8 (10.8%)	2 (2.7%)	0 (0.0%)	3 (4.1%)	22 (29.7%)	74 (8.4%)
61 and above	27 (49.1%)	2 (3.6%)	3 (5.5%)	0 (0.0%)	3 (5.5%)	1 (1.8%)	19 (34.5%)	55 (6.2%)
Total	433 (49.0%)	6 (0.7%)	50 (5.7%)	18 (2.0%)	21 (2.4%)	40 (4.5%)	315 (35.7%)	883 (100.0%)

## Discussion

The success of any medical intervention, including mental health treatment, depends largely on patient adherence to the prescribed regimen. This study explored the treatment non-adherence patterns and discontinuation rates among patients with different mental illnesses.

In this study, the mean age of the patients was 32.8±15.4 years, which was consistent with the results of a previous study by Banerjee and Varma, where the mean age of the participants was 40.0±11.0 years [16]. Out of a total of 883 recruited patients, the majority (60.6%; n=535) were male, and 39.4% (n=348) were female. Most of the study participants were from rural regions.

We found that the treatment-seeking effort was highest among the age group of 21-30 years (31.8%; n=281).

Our findings offer insights into the distribution of diagnoses according to gender. For example, SUDs exhibited a notably higher prevalence (94.6%; n=70) among male patients. Previous studies have reported that men exhibit a higher tendency for regular alcohol consumption compared to women [30]. Similarly, SMDs were more prevalent among male patients, accounting for 76.0% (n=76) of cases. A similar trend was observed in CMDs, which were found to be more prevalent among male patients (56.4%; n=308). Predominantly, it was the male population who accessed the services. There is always a possibility that the females were not able to access the services for more than one reason. Contrary to our study findings, Sriramulu et al., identified a higher prevalence of CMDs among female patients [5]. Similarly, in a study conducted by Sathyanarayana Rao et al., it was also observed that the occurrence of anxiety and depressive disorders was higher in females in comparison to males [31].

When analyzing the prevalence of different diagnoses across the entire patient population, CMDs emerged as the most prevalent, representing 61.8% (n=546) of cases. Interestingly, patients with SUDs as their primary diagnosis represented a small proportion, comprising only 8.4% (n=74) of cases, which is significantly less in comparison to the existing literature. Compared to our study, in a previous investigation carried out by Sriramulu et al., 29.7% of patients had a primary diagnosis of SUD [5]. This difference may be the result of the majority of our study subjects being predominantly Muslims belonging to rural regions; as per available research findings, SUDs are relatively very low among Muslim populations [32].

Our findings suggest that young adults in the 21-40 years, age group were more likely to establish a consistent visiting pattern as compared to individuals in their 50s, who were more likely to discontinue their treatment after the first interaction. Contrary to our study, the majority of previous studies involving individuals with mental illness found that younger age was a prominent factor contributing to treatment non-adherence [33,34].

In our study, women exhibited higher levels of non-adherence when compared to men. The existing literature on this issue lacks uniformity. Similar to our results, a study by Banerjee and Varma, found that women exhibited a greater rate of treatment non-adherence as compared to males [16]; another study also documented higher treatment compliance among male participants [35]. However, other studies have reported conflicting results, where males were more likely to terminate therapy without a doctor’s approval [15,36]; and other studies from India also documented that women were more consistent in their treatment, with a lower dropout rate compared to men [5,19].

The current study also revealed that out of the total patient population of 883, only 35.7% (n=315) were consistent in adhering to treatment over a duration exceeding one year. These results are consistent with those of other studies, which showed that 29.7% of patients were consistent with their treatment for over a year [13]. Similarly, in the above-mentioned study by Banerjee and Varma, 33.1% of patients were adherent to their psychiatric treatment plan [16].

In this study, a significant proportion of patients (49.0%; n=433) discontinued treatment after their initial contact. Furthermore, patients with SUDs exhibited the lowest treatment adherence rate, with only 16.2% (n=12) adhering to their prescribed regimen. Previous research suggests that dropout tends to occur much sooner in treatment (i.e., within the first two sessions) in those with co-occurring alcohol and other SUDs [33,37]. Henzen et al. reported that dropout occurs most frequently within the first two visits for treatment [34]. According to a study by Reneses et al., treatment discontinuation was highest among those with SUDs [38].

A significantly large number of the patients exhibited inadequate adherence to their mental illness treatment. Our results showed that 64.3% (n=568) of patients discontinued their treatment within a period of 1 week to 12 months. This result was in line with those of another study, wherein non-adherence to mental health therapy was found to be 66.9% [16]. In another study, by the end of one year, 71.2% of individuals had stopped receiving treatment for their mental illness [17].

In agreement with previous research findings, our study found that patients with SMDs displayed a better treatment adherence rate of 46.0% (n=46) as compared to other categories. Jain et al. actually reported that the presence of SMDs is a predictive indicator of lower dropout rates [19].

This study did have some limitations. We were not able to include 307 patients from the records because there was no ICD-10 diagnosis available. This constraint led to a reduced patient population, potentially impacting the precision of the results. Furthermore, owing to its retrospective nature, this study could not involve direct patient interaction, which may have impeded the identification of the true causes of their treatment non-adherence. This study is unable to tell whether the patients sought treatment elsewhere (in government or private setup), and the change in providers might not have necessarily meant stopping treatment. As the data were obtained from a single center, we cannot extrapolate the results to the entire country of India. To validate the patterns discussed in this work, future research should gather data from several other centers.

## Conclusions

This retrospective study showed that a significant proportion of patients discontinued their treatment immediately after their initial encounter with the facility. Poor adherence to prescribed treatment in an outpatient clinical setting appears to be a prevalent problem amongst patients diagnosed with mental illness, which is characterized by treatment termination without consulting with their treating doctor. For psychiatric patients who seek treatment, there is a considerably high rate of early treatment dropout. This treatment discontinuation may be avoidable, as the individual identities of these patients are well-known to the provider or facility, and they have had at least one interaction with the healthcare provider. Depending on the outcomes of this investigation, further work must be done to improve treatment adherence among psychiatric patients. Various measures have been proposed with the aim of enhancing adherence to treatment regimens and improving the management of mental health conditions. Additional personnel should be hired to improve patient contact and help them to adhere to their treatment plans. The problem of treatment non-compliance in behavioral health services at mental health facilities can be addressed by examining the coordination of behavioral health services at primary healthcare facilities, adopting the home visit approach, using tracking and tracing strategies, and implementing appropriate technology. The implementation of a patient-centered treatment engagement service is necessary in order to effectively address issues related to dropout rates and reasons for discontinuation within the treatment process. The findings of this research highlight the importance of conducting comprehensive investigations into the factors that can predict disengagement from treatment. Further multicentric studies in this particular domain should attempt to examine the factors that can be indicative of individuals’ willingness to actively participate in therapy.
